# Advanced molecular pathology for rare tumours: A national feasibility study and model for centralised medulloblastoma diagnostics

**DOI:** 10.1111/nan.12716

**Published:** 2021-05-02

**Authors:** Stephen Crosier, Debbie Hicks, Edward C. Schwalbe, Daniel Williamson, Sarah Leigh Nicholson, Amanda Smith, Janet C. Lindsey, Antony Michalski, Barry Pizer, Simon Bailey, Nick Bown, Gavin Cuthbert, Stephen B. Wharton, Thomas S. Jacques, Abhijit Joshi, Steven C. Clifford

**Affiliations:** ^1^ Newcastle University Centre for Cancer Wolfson Childhood Cancer Research Centre Translational and Clinical Research Institute Faculty of Medical Sciences Newcastle University Newcastle upon Tyne UK; ^2^ Newcastle upon Tyne Hospitals NHS Foundation Trust Newcastle upon Tyne UK; ^3^ Department of Applied Sciences Northumbria University Newcastle UK; ^4^ Department of Haematology and Oncology, Great Ormond Street Hospital for Children NHS Foundation Trust London UK; ^5^ Department of Haematology and Oncology Alder Hey Children's Hospital Liverpool UK; ^6^ Sheffield Institute for Translational Neuroscience Sheffield University Sheffield UK; ^7^ Developmental Biology & Cancer Department UCL GOS Institute of Child Health London UK

**Keywords:** molecular pathology, pathology review, biomaterial, biomarkers, diagnostics

## Abstract

**Aims:**

Application of advanced molecular pathology in rare tumours is hindered by low sample numbers, access to specialised expertise/technologies and tissue/assay QC and rapid reporting requirements. We assessed the feasibility of co‐ordinated real‐time centralised pathology review (CPR), encompassing molecular diagnostics and contemporary genomics (RNA‐seq/DNA methylation‐array).

**Methods:**

This nationwide trial in medulloblastoma (<80 UK diagnoses/year) introduced a national reference centre (NRC) and assessed its performance and reporting to World Health Organisation standards. Paired frozen/formalin‐fixed, paraffin‐embedded tumour material were co‐submitted from 135 patients (16 referral centres).

**Results:**

Complete CPR diagnostics were successful for 88% (120/135). Inadequate sampling was the most common cause of failure; biomaterials were typically suitable for methylation‐array (129/135, 94%), but frozen tissues commonly fell below RNA‐seq QC requirements (53/135, 39%). Late reporting was most often due to delayed submission. CPR assigned or altered histological variant (vs local diagnosis) for 40/135 tumours (30%). Benchmarking/QC of specific biomarker assays impacted test results; fluorescent in‐situ hybridisation most accurately identified high‐risk *MYC*/*MYCN* amplification (20/135, 15%), while combined methods (*CTNNB1*/chr6 status, methylation‐array subgrouping) best defined favourable‐risk WNT tumours (14/135; 10%). Engagement of a specialist pathologist panel was essential for consensus assessment of histological variants and immunohistochemistry. Overall, CPR altered clinical risk‐status for 29% of patients.

**Conclusion:**

National real‐time CPR is feasible, delivering robust diagnostics to WHO criteria and assignment of clinical risk‐status, significantly altering clinical management. Recommendations and experience from our study are applicable to advanced molecular diagnostics systems, both local and centralised, across rare tumour types, enabling their application in biomarker‐driven routine diagnostics and clinical/research studies.

## Graphical abstract

Application of advanced molecular pathology in rare tumours is hindered by low sample numbers, access to specialised expertise/technologies, and tissue/assay QC and rapid reporting requirements. We undertook a UK‐wide trial in medulloblastoma (<80 UK diagnoses/year) to establish, and objectively assess, the performance of real‐time centralised pathology review (CPR), incorporating delivery of molecular diagnostics and assessment of genome‐wide technologies (RNA‐seq/DNA methylation array). We demonstrate establishment of national CPR is feasible and enables robust diagnosis to World Health Organisation standards and subsequent risk‐stratification, and highlight essential elements in the implementation of centralised pathology and diagnostics pathways, which are critical to their establishment.

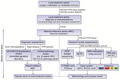

## INTRODUCTION

Advanced upfront molecular diagnostics and pathology review are an increasing requirement in contemporary cancer care and clinical trials. As next‐generation technologies such as high‐throughput sequencing and DNA methylation‐array profiling unearth novel and clinically significant insights in the research setting, their adoption into routine diagnostics and/or standard clinical care is imperative.

Diagnostics delivery in rare tumour types (typically <5 patients per 100,000 population per year^1^) presents specific and significant challenges. Care pathways for such diseases vary with clinical setting but are commonly characterised by systems involving low patient numbers spread across multiple treatment centres. In such settings, co‐ordinated multicentre approaches offer opportunities to address issues including access to specialist analytical technologies and expertise, analysis of tumour and associated biomaterials across multiple centres, tissue/assay quality control and standardisation, requirements for integrated diagnoses encompassing molecular genetic analysis and histopathological review and prompt reporting to accredited standards. For many tumour types, these processes must be completed prior to the commencement of adjuvant therapy, typically within an approximately 30‐day window following primary surgical resection or biopsy.

Centralised diagnostics pathways offer significant potential to address these issues. However, the implementation and performance of centralised molecular pathology review (CPR) systems for rare tumours, and their clinical impact, have not been widely or systematically assessed. Such trials will be essential to support the clinical adoption of such schema and to generate experience which can be applied more widely in routine diagnostics.

Medulloblastoma is an important example of a rare tumour type where molecular advances are rapidly informing its diagnostic requirements and treatment. Medulloblastoma is a clinically and molecularly heterogeneous embryonal tumour arising in the cerebellum, which accounts for ~10% of all childhood cancer deaths. Until recently, medulloblastoma was classified solely by its clinically relevant histological variants, based on morphologic and immunocytochemical criteria; desmoplastic/nodular (DN), medulloblastoma with extensive nodularity (MBEN), classic (CLA), large cell and anaplastic (LCA).^2^ In 2012, international consensus was reached on the definition of four primary medulloblastoma molecular subgroups—WNT and SHH (each named by the predominant mutated molecular pathway that drives their development), Group3 and Group4. Each group is defined by distinct genome‐wide gene expression and DNA methylation patterns and harbours distinct subtypes with characteristic clinical and biological features.^3^, ^4^, ^5^ Moreover, a series of diagnostic and prognostic biomarkers, with established clinical relevance, have now been identified and validated, showing consistent clinical behaviour across multiple studies. These include nuclear accumulation of β‐catenin and activating mutations in its coding gene, *CTNNB1*, and monosomy of chromosome 6—as biomarkers of favourable‐risk WNT subgroup disease.^6^ Validated high‐risk biomarkers include *MYC* or *MYCN* amplification, LCA pathology and *TP53* mutation (in SHH tumours).^3^, ^7^, ^8^

Importantly, medulloblastoma molecular biomarkers (molecular subgroups, *TP53* status) were incorporated, alongside histological variants, into the World Health Organisation (WHO) 2016 classification of central nervous system (CNS) tumours.^2^ Moreover, these are now being used alongside clinical indices to deliver risk‐adapted and targeted treatment strategies in first molecularly driven medulloblastoma clinical trials (NCT02066220, NCT01878617), aimed at improving outcomes and reducing late‐effects. Early studies of the targeted inhibition of the SHH pathway^9^ are in progress. The introduction and rapid upfront assessment of contemporary biomarkers is, thus, essential for entry of medulloblastoma patients into international biomarker‐driven trials, and their adoption as ‘standard of care’. Finally, exemplifying requirements in many rare tumour types, the assessment, cross‐validation and definition of optimal methodologies for detection of specific biomarkers (e.g. *MYC*/*N* amplification, WNT subgroup status), including the relative performance of conventional and next‐generation technologies, is a critical requirement in their standardisation and clinical adoption.

We assessed whether the introduction of national real‐time CPR for medulloblastoma in the United Kingdom would lead to the effective and robust delivery of essential contemporary diagnostics and consequent improved clinical stratification. The annual incidence of medulloblastoma in the United Kingdom is ~80; patients are diagnosed and treated across over 16 specialised treatment centres associated with the UK Children's Cancer and Leukaemia Group (CCLG; https://www.cclg.org.uk/). In the United Kingdom, virtually all CNS tumour diagnosis is undertaken by specialist neuropathologists (with evidence of specialist training in brain tumour diagnosis and on‐going expertise demonstrated by external quality assurance), according to ISO15189 standards within the UKAS (United Kingdom Accreditation Service) framework; this national network, thus, offers a unique opportunity to assess the experience of CPR implementation in practice.

We, therefore, undertook a national multicentre feasibility study of real‐time medulloblastoma CPR, to establish a routine diagnostics infrastructure on behalf of all UK CCLG centres and to assess the experience and impact of CPR, incorporating next‐generation technologies for biomarker assessment. We systematically assessed CPR in 135 patients, demonstrating that it is feasible, provides robust diagnostics to WHO standards and significantly alters clinical management in the setting of initial local diagnosis followed by referral to a national reference centre (NRC). Based on our findings and experience, we proffer recommendations generalisable to all rare tumours, and different diagnostic systems. These should help enable optimisation of pathology review and molecular diagnostics, recruitment to clinical trials and delivery of high‐quality surplus tumour material suitable for comprehensive molecular research studies in these diseases.

## MATERIALS AND METHODS

### Study design

**FIGURE 1:**
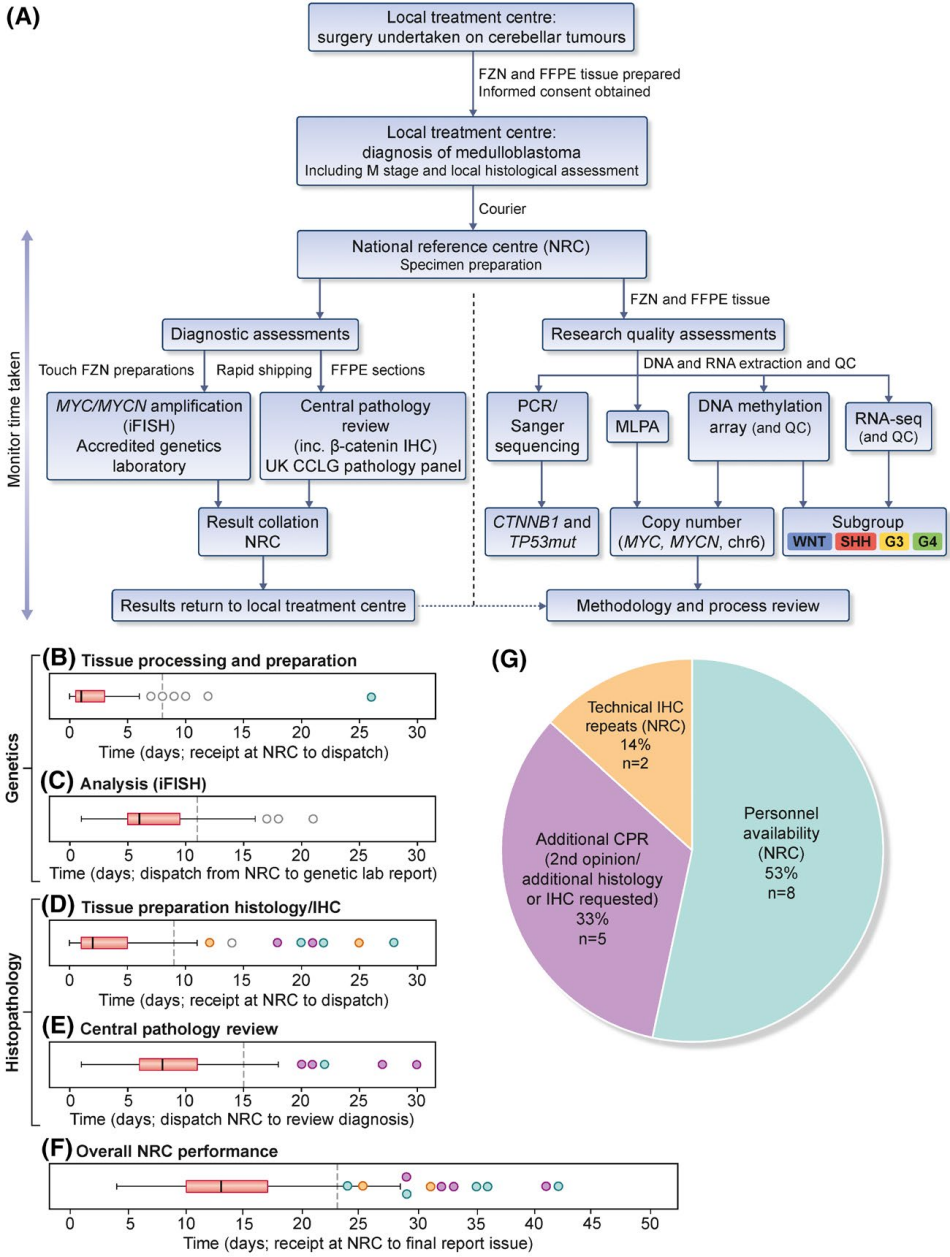
A UK national trial of centralised real‐time medulloblastoma molecular diagnostics: Study workflow, specimen handling and turnaround times. (A) Study overview. Molecular subgroups are shown in their consensus colours WNT, blue; SHH, red; Group3, yellow; Group4, green. Boxplots showing turnaround times (days) for (B) iFISH tissue processing and preparation and (C) iFISH analysis and reporting. Boxplots showing turnaround times (days) for (D) histopathology tissue preparation/IHC and (E) central pathology review and reporting. (F) Overall turnaround time from receipt in the NRC to issue of final report to local centre. Dotted lines represent mean turnaround time +1 SD. Cases that exceeded the overall turnaround threshold of mean +1 SD (F) were coloured according to the reason for delay; technical IHC repeats (black), additional CPR (blue) and NRC personnel availability (green). Proportion of cases assigned to category are shown in (G). Abbreviations: FZN, Frozen tissue; FFPE, formalin‐fixed paraffin‐embedded; QC, quality control; MLPA, multiplex ligation‐dependent probe amplification

This link‐anonymised national trial was set up to investigate the feasibility of real‐time medulloblastoma diagnostics within a clinically defined timeframe (i.e. before the start of adjuvant therapy) (Figure 1A). Diagnostic assessments (fluorescent in‐situ hybridisation [iFISH] for *MYC*/*MYCN* amplification status, β‐catenin immunohistochemistry [IHC]), undertaken in laboratories accredited under UKAS to International Standards (ISO 15189:2012), along with central pathology review, were performed in real time with the intention of reporting results within a 30‐day period. Research assessments (mutational analysis for *CTNNB1* and *TP53*, molecular subgroup analysis by 450k methylation‐array and RNAseq and copy number calling by multiplex ligation‐dependent probe amplification (MLPA) and methylation‐array) were performed post hoc on DNA and RNA derived from frozen study samples to evaluate sample handling and DNA/RNA quality.

### Patient recruitment, registration, sample shipping and reporting

The study was open to all UK patients from 2009 to 2015. 16 UK CCLG treatment centres submitted eligible patients defined as: any patient with a local diagnosis of MB for whom both formalin‐fixed, paraffin‐embedded (FFPE) and frozen tissue tumour material were available (*n* = 135, median age at diagnosis, 7.59 years). Submitting centres were required to register patients and send tumour material to an NRC at the Newcastle on Tyne NHS Hospitals Trust/Newcastle University, according to standard operating procedures (SOPs; Supporting Information online). An approved courier was used for overnight transport of human tumour samples from the local centre to the NRC on dry ice (frozen tissue) or at ambient temperature (FFPE tissue). Time taken for shipment, tissue processing and reporting of centralised diagnostic assessments (i.e. central pathology review and molecular testing) was recorded. Diagnostic results were not reported back to submitting centres under the terms of study ethics approval; however, technical metrics (success or failure) were reported.

### Diagnostic assessments

#### Formalin‐fixed, paraffin‐embedded histology

For immunohistochemistry [IHC], histology tissue sections of 4 μm were cut on a rotary microtome and mounted onto Superfrost plus slides (Thermo Scientific, 1014356190) and dried at 60°C for 1 hour. H&E was carried out to standard protocols and reticulin was stained according to the Gordon and Sweet method^10^ (Supporting Information online). A medulloblastoma diagnostic IHC panel was applied to premounted slides with their appropriate control sections using the fully automated Ventana BenchMark XT IHC system (Supporting Information online).

#### Central pathology review

Stained slides from each patient were assigned to one of four pathologists from the UK Children's Cancer and Leukaemia Group (T.S.J., K.R., S.B.W. and A.J.), and centrally reviewed according to WHO 2007 criteria.^11^ Tumours were classified into the following histological variants; CLA (9470/3), LCA (9474/3), DN (9471/3) or MBEN (9471/3). Local pathologists could elect not to define variant in anticipation of assignment by CPR. For cases where the CPR variant call was discrepant to the local call, each remaining pathologist was requested to additionally review, before consensus on histopathological variant classification was reached between pathologists. Tumour cell content was assessed in all cases.

#### β‐catenin immunohistochemistry scoring

All 16 samples that were either mutant for *CTNNB1* or positive for β‐catenin IHC nuclear localisation (≥10% of cells) on initial analysis were sent for blinded, independent, review to three neuropathologists (S.B.W., T.S.J., K.R.), alongside 16 samples that were *CTNNB1* wild‐type or showed β‐catenin IHC nuclear localisation in <10% of cells. All samples were rescored; consensus was defined based on agreement of ≥2 neuropathologists.

#### Nucleic acid extraction

DNA was isolated from fresh frozen and FFPE primary tumour biopsy material according to established methods (Supporting Information online).

#### Fluorescence in‐situ hybridisation

Frozen tissue was assessed for tumour content by H&E staining of 8 μm frozen sections; touch imprints were prepared from the exposed face and then interphase FISH for *MYC*/*LAF* and *MYCN*/*IGH* was carried out using standard protocols (Supporting Information online). *MYC*(*N*) amplification scoring was as per Ryan et al.^12^ and the SIOP‐PNET5‐MB protocol; scoring was undertaken by two independent assessors, as previously described. ^13^, ^14^

### RESEARCH QUALITY ASSESSMENTS

#### Mutational analysis

*TP53* (exons 4–9) and *CTNNB1* (exon 3) mutation status was assessed by direct polymerase chain reaction (PCR)–based DNA sequence analysis according to the protocols described in Hill et al.^15^ and Ellison et al.^16^

#### Molecular subgroup assessment

Intact DNA of >500 ng, in a total volume of <45 μl, was suitable for methylation‐array (Illumina). RNA quantity and quality was assessed by Agilent 2100 Bioanalyser and those RNAs >800 ng and with an RNA integrity number (RIN) >5 were suitable for RNAseq and subsequent molecular subgrouping. Subgrouping according to methylation and expression profiles was achieved using established methods.^3^ SHH and Group3/Group4 second‐generation subtypes were assigned according to the ‘Grp3 and Grp4 Classifier’ found at https://www.molecularneuropathology.org/mnp/classifier/7.^5^

#### Copy number estimation

As well as iFISH (see above), copy‐number for *MYC(N)* was estimated by 1) MLPA and 2) (additionally for chromosome 6) 450 k array probe intensity values using the R package conumee,^17^ as previously described.^18^ Copy‐number assessment by MLPA (using SALSA reagents; MRC‐Holland) for *MYC(N)* were as described^15^ and measured relative to four independent reference loci (*B2* *M*, *TBP*, 7q31 and 14q22).

For further detail, see Supporting Information online.

## RESULTS

### Turnaround times

A total of 135 samples were received by the NRC from 16 treatment/referral centres. The mean and median interval between date of surgery and arrival at the NRC was 24 and 14 days, respectively. The range of 1–301 days reflected the study protocol; specifically, guidance was issued that allowed samples to be registered and submitted without specific time limits, to maximise cohort size and assessment of NRC performance.

A total of 127/135 (94%) samples were processed for iFISH within 8 days of receipt by the NRC (a calculated threshold, defined as the mean processing time plus 1 standard deviation (SD); dashed line Figure 1B). iFISH analysis and reporting was completed within 11 days of dispatch from the NRC (mean + 1 SD) for 130/135 (96%; Figure 1C). A total of 121/135 (90%) of tissues were processed for histology/IHC within 9 days (mean + 1 SD; Figure 1D), and subsequent central pathology review was completed within 15 days for 94% (127/135). Overall, results for 120/135 (88%) of cases were reported to the submitting local centre within 23 days of receipt by the NRC (Figure 1F). For those for which the mean + 1 SD time threshold was exceeded (15/135; 12%), reasons included personnel unavailability (53%; 8/15), additional CPR (second opinion, additional requested histology and/or IHC; 5/15 [33%]) and technical IHC repeats required within the NRC (2/15; 14%; Figure 1G). The mean and median intervals between date of surgery and final reporting was larger, at 40 and 28 days, respectively (range 14–332 days), reflecting the permissive recruitment approach with regard to patient registration and sample submission to the NRC.

### Sufficiency of biological material to support contemporary diagnostics and next‐generation analyses

**FIGURE 2:**
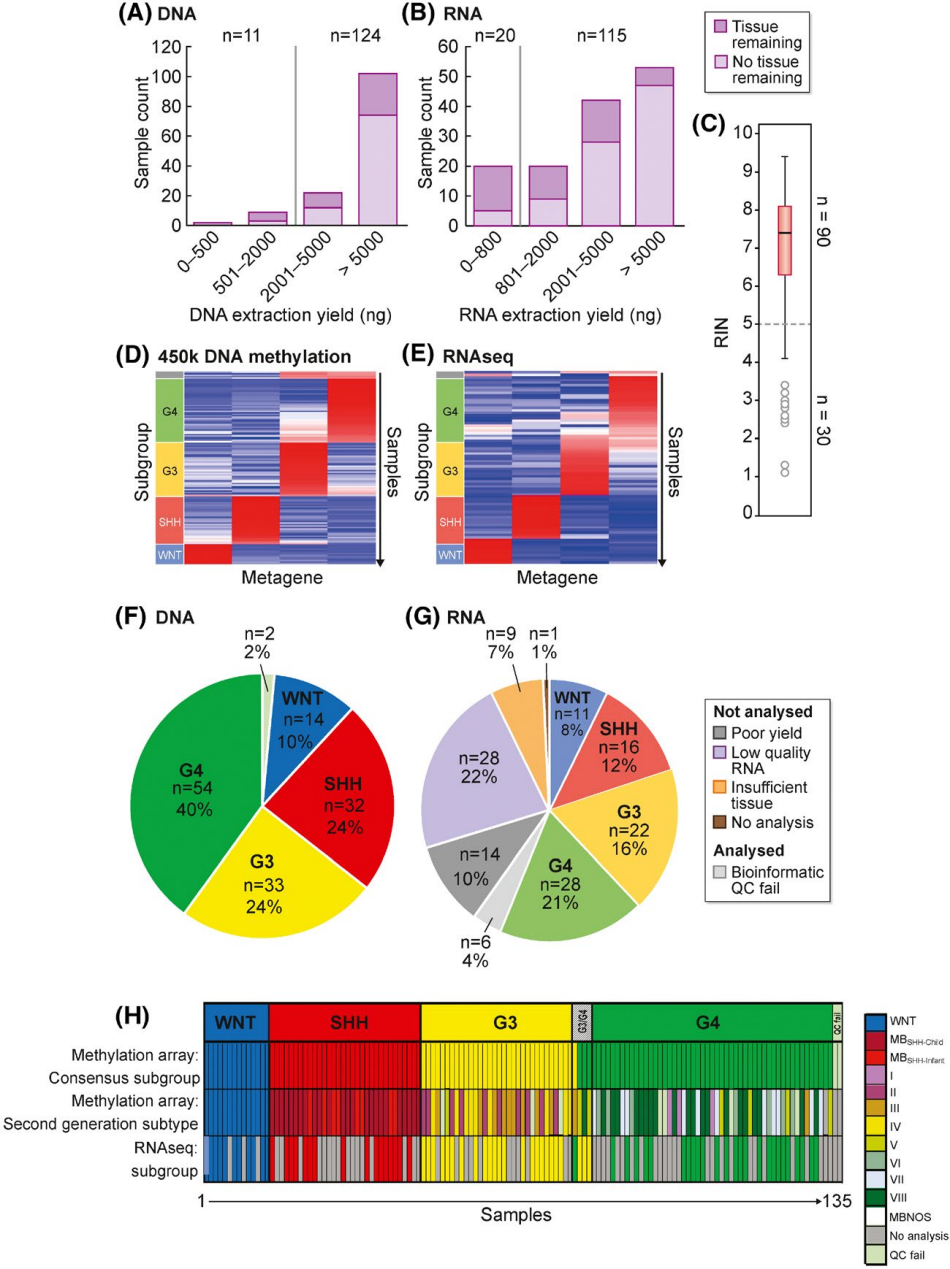
Next‐generation molecular diagnostics: quality control and suitability of extracted nucleic acids for molecular subgrouping. Bar plots of DNA (A) and RNA (B) extraction yields from tumour material. Light purple, no remaining tissue postnucleic acid extraction; dark purple, remaining tissue. Red lines represent typical quantity thresholds that must be exceeded to allow for downstream research assessments including molecular subgrouping. (C) RIN assessment of RNA quality. Red line represents a quality threshold of 5, below which samples are ineligible for RNAseq. Non‐negative matrix factorisation (NMF) clustering of (D) DNA methylation profiles and (E) expression profiles. Pie charts summarise molecular subgroup assignments derived from (F) DNA methylation‐array or (G) RNAseq. Failed samples are shown in shades of grey; reasons for failure are poor nucleic yield, low quality RNA (RIN < 5) and insufficient tissue. H, Molecular subgroup assignment by DNA methylation‐array (consensus 4‐subgroup assignment and second‐generation subtype) and RNAseq (consensus 4‐subgroup assignment) [Correction added on 31 May 2021, after first online publication: Panel (C) was mislabelled previously and has been added in this version.]

We examined whether current tissue handling practices across UK treatment centres were sufficient to provide the abundant high‐quality tumour material typically required for contemporary diagnostics and research methodologies. The majority (102/135; 76%) of frozen tissue samples yielded more than 5000 ng of dsDNA; 73% of those had surplus tissue remaining for further extraction. Furthermore, 124/135 (92%) exceeded a threshold of 2000 ng dsDNA (red line Figure 2A), which allowed for all real‐time diagnostic and *post hoc* research methodologies to be carried out (Sanger sequencing, methylation‐array and MLPA). Conversely, RNA was extracted from 126/135 (93%) of frozen tissues with failures due to sample limitations. 116/125 of attempted RNA extractions yielded more than a threshold amount of 800 ng (Figure 2B, red line), necessary for RNAseq. RNA quality was assessed by the RIN; 73% (91/125) of those measured had RIN>5, indicating the RNA was of sufficient quality for RNAseq (Figure 2C).

### Molecular subgrouping

Overall, 133/135 (99%) samples yielded DNA of sufficient quality and quantity for methylation‐array; 81/135 (60%) were suitable for RNAseq. Non‐negative matrix factorization (NMF) was used to cluster cohort methylation^3^ (Figure 2D) and expression profiles (Figure 2E). Four subgroup‐specific metagenes were defined; a support vector machine (SVM) classifier assigned subgroups (WNT, SHH, Group3 and Group4). Samples classified with <80% confidence (by resampling procedures) were deemed to be non‐classifiable (NC).^5^

Robust consensus subgroup assignment (>80% confidence) was achieved for 129/132 (98%) DNA methylation profiles; Group3/Group4 second‐generation subtypes recently defined by Sharma et al. within Group3 and Group4^5^ were confidently assigned for 128/132 (97%) and showed the expected distribution (subtype VIII predominated in Group4; II, III and IV were the majority subtypes in Group3; Figure 2H). Success rates were comparable for RNAseq‐based subgroup assignment (75/81, (93%); Figure 2F,G), demonstrating that when DNA and RNA entry criteria thresholds for sample requirements are consistently applied (e.g. concentration, RIN), samples rarely fail to exceed 80% confidence estimates for subgroup assignment.

### CPR is critical for robust diagnosis of histological variants

**FIGURE 3:**
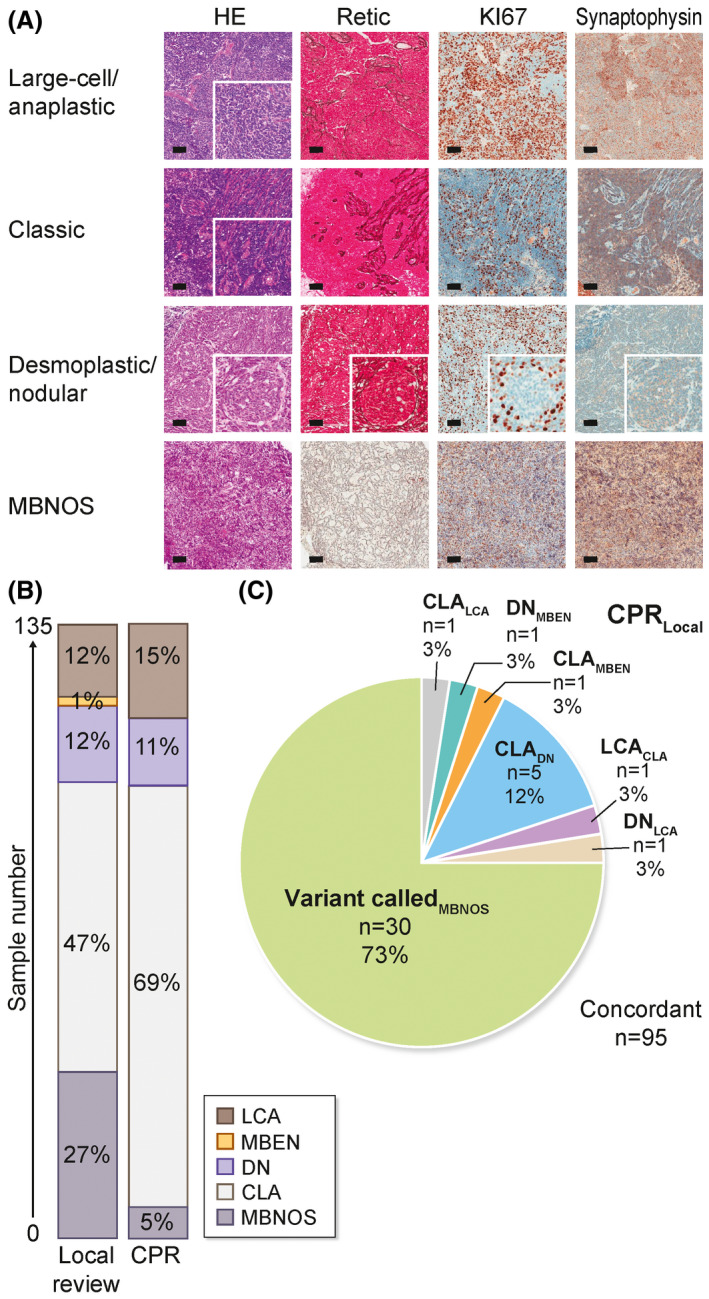
Central pathology review (CPR): concordance with local pathology assessment. A, Representative micrographs to show the WHO (2007) histological variants assessed in this study; large cell/anaplastic, CLA, DN and MBNOS. No tumours were classified as MBEN by CPR. Magnifications of ×20 are shown. Scale bar = 200 μm. B, Stacked bar plot showing proportion of pathological variants assigned by local review vs CPR. Key shows colours used to denote pathological variants. C, Pie chart to show the nature of revised pathology calls between local (subscript text) and CPR. Abbreviations: HE, haematoxylin and eosin (gross morphology); Retic, reticulin (marker of desmoplasia); Ki‐67 (marker of proliferation); CLA, classic; DN, desmoplastic/nodular; MBEN, medulloblastoma with extensive nodularity; LCA, large cell/anaplastic; MBNOS, medulloblastoma not otherwise specified

Following diagnosis at their local treatment centre, all samples were reviewed centrally by a panel of three experienced neuropathologists, according to the 2007 WHO classification of tumours of the CNS (Figure 3A).^11^ Tumours that could not be classified histologically according to this scheme were termed medulloblastoma, not otherwise specified (MB‐NOS).

All submitted tumours were diagnosed locally as medulloblastoma; 105/135 (78%) had a pathology variant assigned locally. CPR changed (10/105, 10%) or assigned (30/135, 20%) pathology variant for 40/135 (30%) of tumours. Reticulin staining was crucial in resolving misclassifications, indeed, reclassification of DN tumours as CLA was the predominant recurrent reclassification (*n* = 5), with other reclassifications occurring infrequently (Figure 3C). Overall, variant frequencies following CPR were CLA (69%; 93/135), LCA (15%, 20/135), DN (11%, 15/35) and MBEN (0%, 0/135). Five percent (7/135) remained without a definitive histological variant post‐CPR, either due to insufficiency of supplied tumour material (*n* = 2) or difficult to interpret complex histologies (*n* = 5). Tumour cell content was assessed on a section of frozen biopsy material as part of CPR and exceeded 60% in all cases (mean, 87%; range 60%–100%).

### Multi‐assay WNT assessment improves subgroup assignment

**FIGURE 4:**
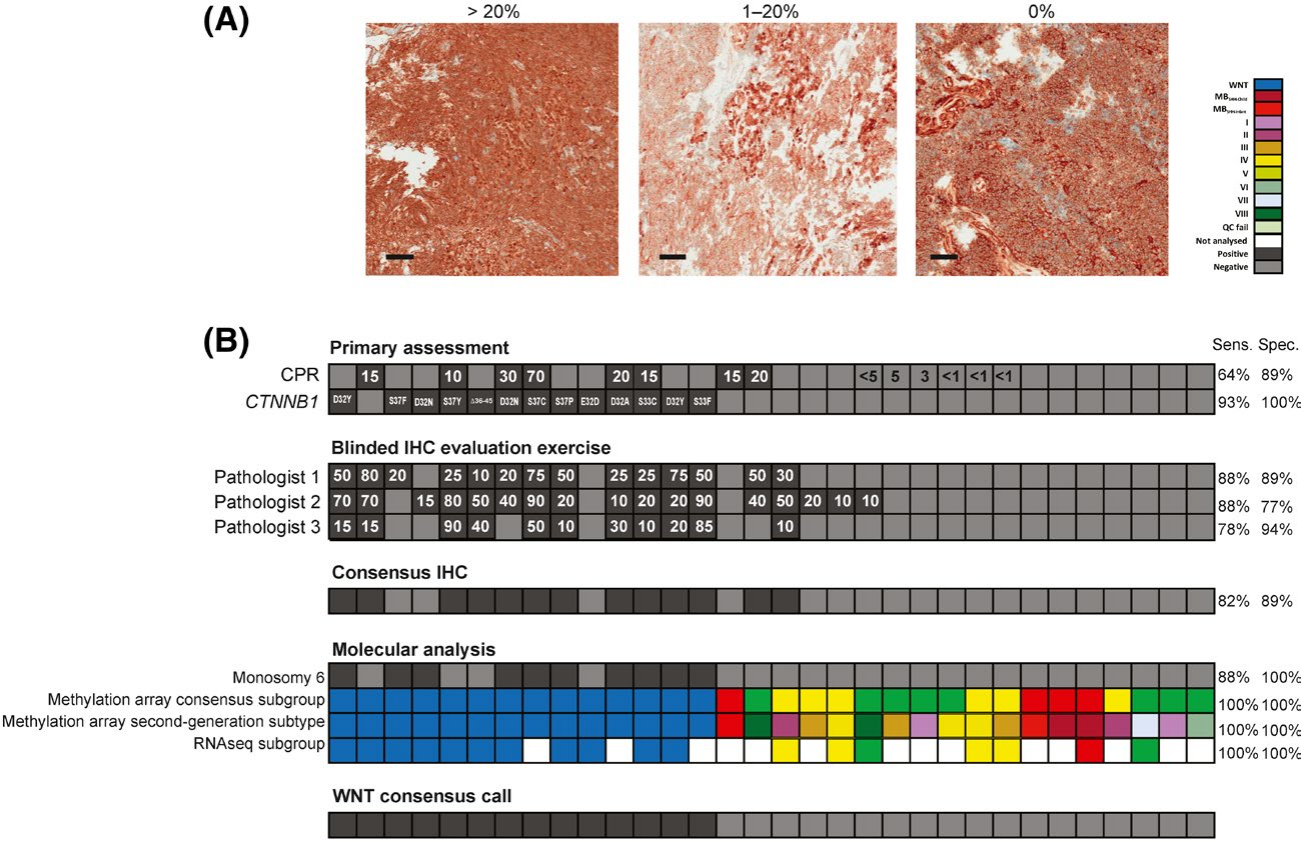
Biomarker assay assessment and validation I: WNT molecular subgroup—comparison of IHC and molecular methods and blinded assessment of interpathologist concordance. A, Illustrative immunohistochemistry (IHC) micrographs showing variation in nuclear β‐catenin accumulation in WNT subgroup cases (>20% and 1%–20%). A non‐WNT subgroup case (0% nuclear β‐catenin accumulation, membranous staining only) is also shown. Magnifications of ×20 are shown, scale bar = 200 μm. B, A comparative analysis of WNT subgroup assignment methods. Primary assessment of WNT status by 1) % nuclear β‐catenin accumulation determined by CPR (white text in black boxes shows % nuclear accumulation >10%, those cases with evidence of nuclear positivity <10% shown in black text) and 2) *CTNNB1* mutation status (amino acid changes given). % nuclear β‐catenin accumulation >10% is also shown for a blinded, three‐pathologist IHC evaluation exercise with consensus between 2 or more pathologists from CPR and the blinded evaluation exercise shown by black shading. Assessment of WNT status by molecular analyses are shown. Cases with monosomy 6 are identified by black shading, DNA methylation‐array (consensus 4‐subgroup, and second‐generation subtype) and RNAseq subgroup calls are shown. An overarching WNT consensus call (defined by either *CTNNB1* mutation status or IHC positivity plus at least one other molecular method) is shown, and each WNT status assignment methods is compared to this consensus (Sens., sensitivity; Spec., specificity). Grey boxes, negative results; white, not done

WNT subgroup status was used as an exemplar to assess inter‐observer variability in IHC interpretation. WNT subgroup medulloblastomas are defined by activation of the Wnt/Wg signalling pathway, associated with nuclear accumulation of the β‐catenin protein, *CTNNB1* mutation and monosomy of chromosome 6.^6^, ^16^ β‐catenin IHC methods have historically been used to evaluate WNT subgroup status, but inter‐observer variability and sample heterogeneity may confound this assessment.^19^ All cases showing any evidence of β‐catenin nuclear accumulation in real‐time CPR (1%–100%; *n* = 14), selected negative controls (n=10) and all *CTNNB1^mut^* cases (*n* = 8) were subjected to a further round of independent, blind, pathology review by all three neuropathologists (total *n* = 32; Figure 4A,B). IHC consensus was defined as agreement between two or more pathologists and concurred with initial CPR in 25/32 (78%) of cases.

We then tested how diagnostic accuracy compared with molecular evaluation; methylation‐array‐based subgrouping, Chr6 monosomy and RNAseq‐based subgrouping. The defining WNT consensus call was determined by either *CTNNB1* ^mut^ or positivity for two or more of the four WNT status measures. Methylation‐array and RNAseq subgroup calls had 100% sensitivity and specificity; Chr6 monosomy had a specificity of 100% and 88% sensitivity. Of the IHC evaluations, consensus 3‐pathologist review status was more sensitive than initial CPR (82% vs 64%), with indistinguishable specificities (89%). Two cases were false positives by primary CPR IHC, compared with the WNT consensus call, and were negative for all other WNT measures.

### *MYC* and *MYCN* amplification assessment: fluorescent *in‐situ* hybridisation is a clinical diagnostic ‘gold standard’

**FIGURE 5:**
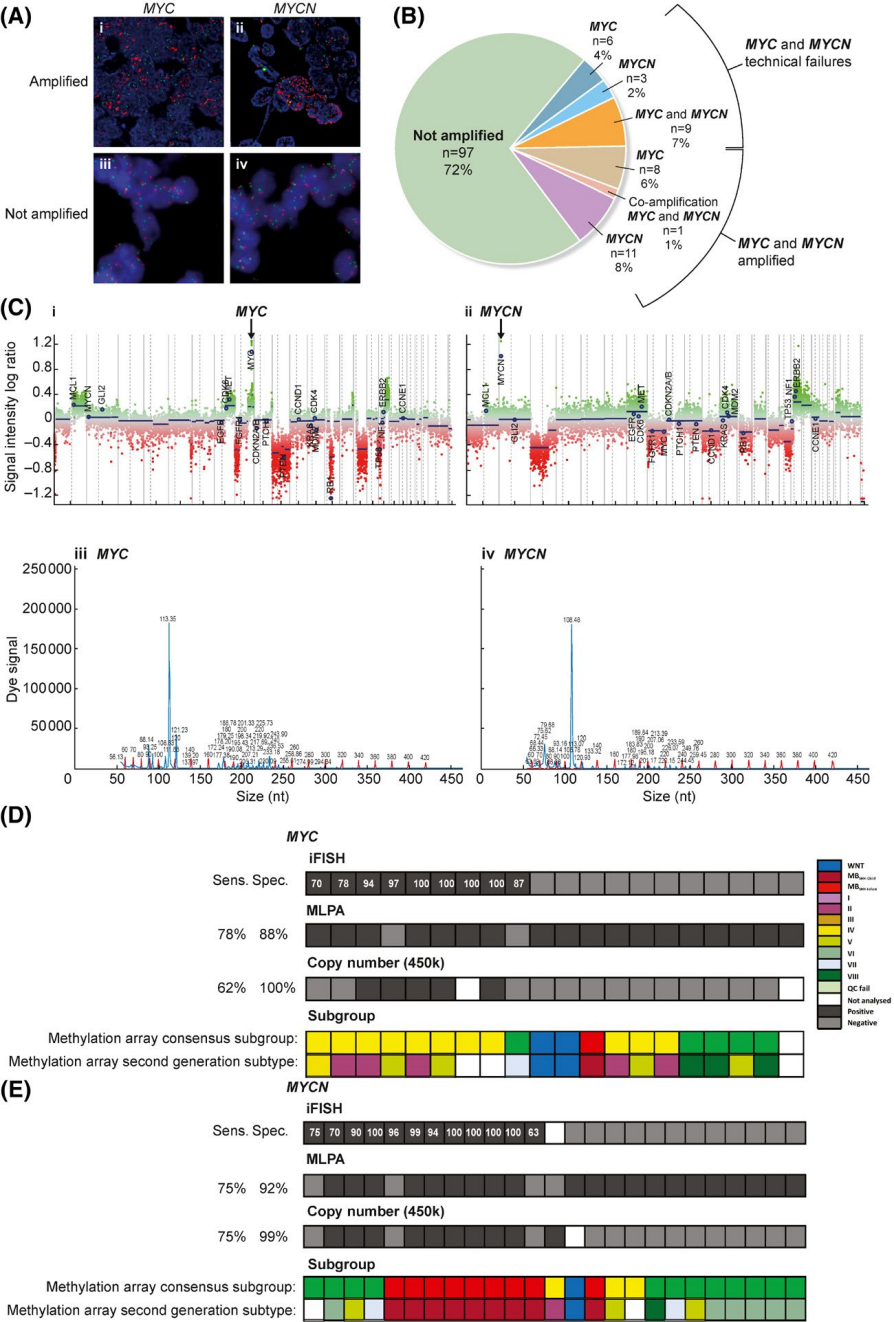
Biomarker assay assessment and validation II: *MYC* and *MYCN* amplification—comparison of iFISH, DNA methylation‐array copy number profiling and MLPA methods. A, iFISH micrographs representing amplified (A I, II) and nonamplified (A III, IV) tumours. Red; *MYC* or *MYCN*, green *LAF4* (for *MYCN*) or IGH (for *MYC*) controls. Bb, Frequency of *MYC* and *MYCN* amplification, and technical failures, across the trial cohort. C, example *MYC* and *MYCN* amplified tumours determined using by DNA methylation‐array (upper panel) or MLPA (lower panel). A comparative evaluation of iFISH *MYC* (D) and *MYCN* (E) status assignment vs MLPA and DNA methylation‐array. Black boxes represent amplification of *MYC/MYCN*; grey boxes, negative for amplification; white boxes, NA. Numbers in iFISH track are % of amplified nuclei. DNA methylation‐array (consensus 4‐subgroup and second‐generation subtype) subgroup calls are shown. MLPA and DNA methylation‐array *MYC* and *MYCN* assignment calls are compared to iFISH (Sens., sensitivity; Spec., specificity)

iFISH was attempted in all samples received (Figure 5A, representative analyses). Out of the 117 (87%) successful *MYC* and *MYCN* iFISH assays, 6% were *MYC* amplified (*n* = 8), and in 9% *MYCN* was amplified (*n* = 12). Technical failures affected 13% (*n* = 18) of the cohort (*MYC*, *n* = 6, 4%; *MYCN*, *n* = 3, 2% or both *n* = 9, 7%; Figure 5B). Due to this failure rate and the increasingly common adoption of alternative methods for *MYC* and *MYCN* amplification status assessment, we undertook CN analysis using DNA methylation‐array^17^ (successful in 133/135) and MLPA^20^ (successful in 124/135, *MYC*; 126/135, *MYCN*; Figure 5C; illustrative examples). Of the nine cases shown to be *MYC* amplified by iFISH, seven also had elevated copy number by MLPA along with 11 false‐positive cases. A total of 88 cases showed no evidence of *MYC* amplification, in agreement with iFISH data (not shown) (overall MLPA sensitivity, 78%; specificity, 88%). DNA methylation‐array analysis showed complete specificity but poor sensitivity (62%; Figure 5D). These observations were recapitulated for *MYCN* amplification analysis, with 9 of the 12 iFISH positive cases also showed elevated copy number by MLPA, but with a number of false positives (*n* = 10; sensitivity, 75%; specificity, 92%; Figure 5E). DNA methylation‐array analysis showed the same sensitivity (75%) but had a no false positives (specificity, 100%). *MYC*‐ and *MYCN*‐amplified cases showed the expected subgroup‐specific distributions; the vast majority (8/9) of *MYC*‐amplified cases were Group3 (Figure 5D), whereas 11/12 *MYCN* amplified cases were SHH (*n* = 6) or Group4 (*n* = 5; Figure 5D,E).

### Application and impact of CPR: diagnosis to SIOP‐PNET5‐MB and WHO (2016) criteria

**FIGURE 6:**
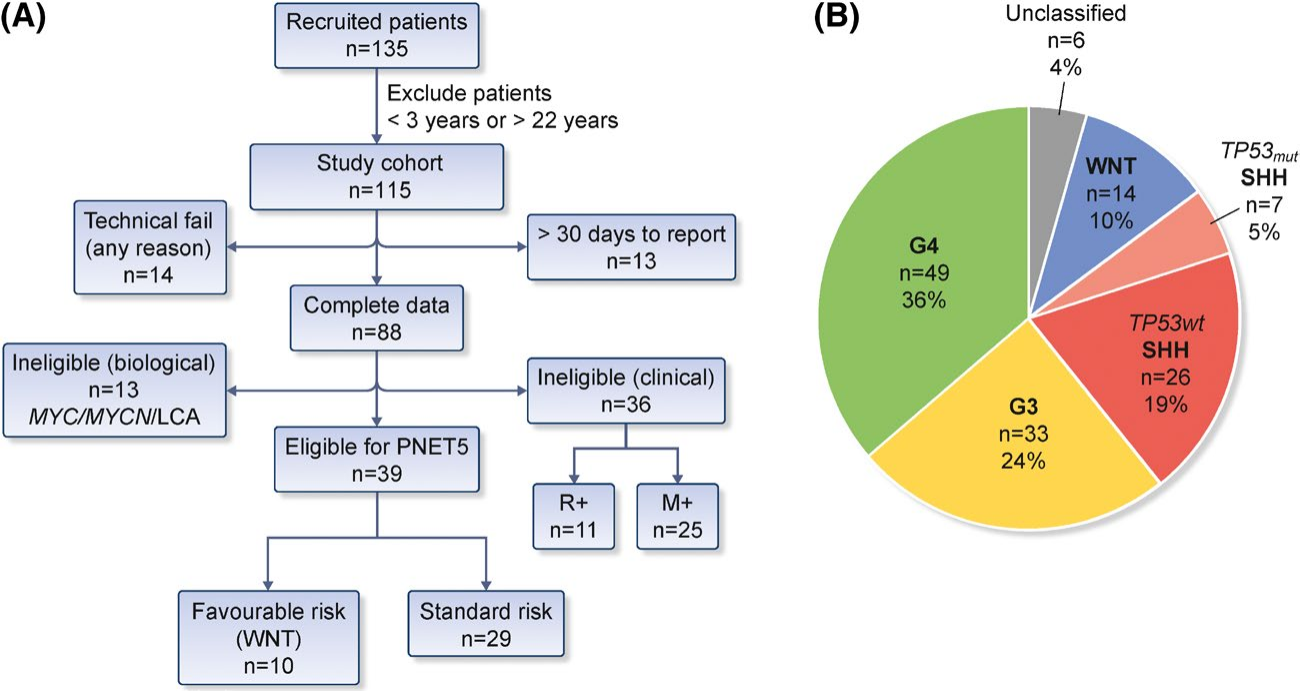
Application and impact of CPR and molecular diagnostics: diagnosis to SIOP‐PNET5‐MB and WHO (2016) criteria. A, Application of SIOP‐PNET5‐MB clinical trial criteria and (B) the 2016 World Health Organization classification of CNS tumours^2^ to our cohort. Abbreviations: LCA, large cell/anaplastic; R+, incompletely resected disease; M+, metastatic disease

The pan‐European SIOP‐PNET5‐MB clinical trial of standard‐risk medulloblastoma (NCT02066220) stratifies medulloblastoma patients from 3–22 years of age at diagnosis upfront using clinical and biological criteria, tested in real time. We applied the SIOP‐PNET5‐MB clinical trial entry criteria to our cohort, to forecast likely recruitment to a risk‐stratified clinical trial (Figure 6A). A total of 115/135 met the age criteria of 3–22 years. Of these 115 patients, 27 (23%) would have been ineligible for entry into the trial. For 14/115 (12%) of these ineligible patients, this was due to potentially avoidable assay failures. Furthermore, 13/115 patients (11%) would have been excluded due a failure to complete pathology review or molecular diagnostics within the interval mandated by SIOP‐PNET5‐MB (we applied a maximum of 10 days for receipt by the NRC, then a cut‐off of a further 28 days for final reporting by the NRC).

In total, there were 88 patients with the complete clinical and biological data required to assess trial entry criteria. Of those 88, 13 showed evidence of high‐risk biological and pathological features (*MYC*(*N*) amplification and/or LCA pathology), which would have rendered them ineligible for the SIOP‐PNET5‐MB. Thirty‐six further patients would have been excluded based on the presence of high‐risk clinical features (incomplete surgical resection, *n* = 11; metastatic disease, *n* = 25), leaving 34% of age‐appropriate patients (39/115) eligible for SIOP‐PNET5‐MB. A total of 26% (10/39) of these were WNT‐positive, compatible with eligibility for the favourable‐risk trial arm, with the remaining 74% (29/39) considered standard‐risk.

The 2016 World Health Organization (WHO) classification of tumours of the CNS recognises five distinct medulloblastoma molecular variants; (1) WNT‐activated, (2) SHH‐activated *TP53* mutant, (3) SHH‐activated *TP53* wild‐type, (4) non‐SHH/non‐WNT (encompassing Group3 and Group4 as provisional variants).^2^ In our cohort, non‐SHH/non‐WNT predominated as expected; 36% (*n* = 49) were Group4 and 24% (*n* = 33) were Group3. Fourteen (10%) WNT patients were identified (Figure 6B). *TP53* mutation status subdivided the SHH group into the respective *TP53* mutant (*n* = 7, 5%) and wild‐type (*n* = 26, 19%) groups. Overall, 96% of our cohort were successfully classified to WHO (2016) standards; this was not possible for the remainder due to a lack of molecular subgroup data.

Taken together, 29% (33/115) of patients had altered risk‐status based on CPR and molecular assessments made (i.e. assignment of *MYC*(*N*), LCA, WNT and SHH‐*TP53* status).

## DISCUSSION

This study has established and implemented centralised real‐time medulloblastoma molecular pathology for the United Kingdom. In this setting within the UK clinical system (i.e. 16 dispersed local treatment centres), CPR at an>a NRC enhanced the diagnostic process through improved histological variant assignment, molecular variant assignment, assessment of risk‐biomarker status including application of next‐generation technologies and consequent assignment of disease risk‐status.

Care and referral systems differ globally, defined by factors including population density, geography and national care systems for specific diseases (e.g. dispersed networks of local treatment centres vs large referral/treatment centres), alongside associated availability of molecular diagnostic technologies. From our experience, we provide recommendations for routine pathology review and molecular diagnostics, which are applicable in different care systems and can inform the design and delivery of current and future diagnostic testing and clinical trials (Table 1). These findings apply not only in medulloblastoma but also for other rare tumours, where collection and rapid assessment of biological material is key.

**TABLE 1:** Recommendations for the delivery of advanced molecular pathology for rare tumours in real‐time, including medulloblastoma‐specific recommendations

Scope	Recommendations
All rare tumours	**Overarching governance**
	Adequate number of well‐trained staff
	Clear and concise SOPs
	**Patient registration**
	Prompt consenting and registration
	**Sampling and sample handling**
	Sufficient FFPE and promptly snap‐frozen tumour tissue (10 mm^3^)
	Prompt transfer from surgical theatre to pathology lab
	Prompt shipping to reference centre/central site/biobank
	**Sample movement logistics (centralised systems)**
	Establishment of appropriate material transfer agreements
	Excellent lines of communication between local and reference centres
	Use of reliable courier
	**Real‐time molecular diagnostics and pathology review**
	Undertake sample quality assurance
	Initiate all molecular testing in parallel to ensure timely completion
	Use robust, validated tests, performed in accredited laboratories
	Pathology review with a multineuropathologist review panel
	Prompt reporting pathways
	**Provision of biomaterials for research investigations**
	Adequate biobanking systems and facilities
	Anonymised coupling of tumour material data to comprehensive patient data
Medulloblastoma	**WNT subgroup assessment**
	*CTNNB1* mutation and DNA methylation‐based classification are robust, sensitive and specific
	Consider monosomy 6 or RNA‐based classification, as supportive assessments
	***MYC*(*N*) amplification assessment**
	iFISH is the diagnostic ‘gold standard’

Abbreviations: CPR, central pathology review; FFPE, formalin‐fixed, paraffin‐embedded; SOPs, standard operating procedures.

Histopathologic and associated review practice also varies significantly internationally. The UK neuropathology community provides a key framework to enable assessment of the value and impact of CPR within a highly specialised group of professionals. In the United Kingdom, neuropathologists qualify to practice following specialist training (assessed by examination) and participate in regular ongoing mandatory national EQA activities (https://www.bns.org.uk/eqa‐scheme/). Therefore, all tumours, prior to CPR, have been diagnosed by a specialist. We observed that, where local neuropathologists had assigned a medulloblastoma histological variant, there was a good degree of concordance with CPR calls, although significant discordances did occur (10/105; 10%); the distinction between certain DN variants and CLA tumours represented the most common discrepancy. Furthermore, in many cases, local neuropathologists did not assign a histological variant and CPR did; this may in part reflect local practice to use CPR for variant calling. Overall, variant was assigned or altered by CPR in 30% (40/135) of patients, strongly supporting the added value of the CPR process. In any central review system, it is essential to have mechanisms in place to identify and examine discrepancies between local and central opinion, and to reach consensus, and the involvement of multiple reference pathologists in the CPR team was critical to this. We further anticipate discordance will be greater in circumstances where brain tumours are assessed by nonspecialist pathologists and/or more dispersed models of practice; based on our experience, we recommend review by multiple expert pathologists as essential in such instances. The introduction of tools to support the diagnosis of histologically defined variants (e.g. image analysis, associated biomarkers) represents a promising area for future development.

Considering sampling and logistical aspects; trained, professionally accredited staff, working to clear and concise SOPs are essential at all stages. These include practice in the surgical theatre, where resource limitations and cultural practices can cause conflicting priorities between resection of the tumour, diagnostics and the ability to sample adequate biomaterials. Later, the processing of samples and the coordination of their pathology review, molecular diagnostics and reporting can also impact. Importantly, we observed a lack of prompt patient registration and shipment of biomaterials to the NRC. Of note, the study protocol did not permit intervention; guidelines were issued, and patient registration/sample submission was allowed to proceed without influence by the NRC. Marked improvements are anticipated in settings where real‐time molecular diagnostics are mandated clinically to defined timescales. Indeed, this has been borne out by the improved submission rates and turnaround times on the SIOP‐PNET5‐MB medulloblastoma trial (NCT02066220), now underway; we estimate that >90% of UK medulloblastomas were submitted to the NRC in 2019. We anticipate the obligation to meet such eligibility criteria will be a strong driver of timely completion of the CPR pathway.

Once samples reached the NRC, outcomes were more consistent. The vast majority (88%) had completed diagnostic assessment within 23 days of receipt at the NRC; delays were typically due to personnel availability, the requirement for repeat analyses and further CPR. Insufficiency of submitted biomaterials was apparent, and we concur with previous recommendations^21^ that a minimum of 1.5 cm^3^ of FFPE tissue and 0.5 cm^3^ of frozen tumour tissue should be collected where possible. However, in this study we found that the quality of frozen tumour material was a greater risk to successful delivery of molecular diagnostics. Although DNA is less sensitive to poor tissue‐handling practices (reflected by the near‐total ability to perform successful DNA methylation array), frozen material submitted was often not suitable for RNA‐seq‐based analysis. Although it is true that RNAseq sets a high bar for quality, and other molecular tests are suitable for use on RNA of lesser quality and/or quantity (e.g. RT‐PCR, nanoString, ISH), or on DNA extracted from FFPE samples (e.g. DNA methylation array and NGS methods), it is nevertheless vitally important that the practice and culture of the operating room and pathology laboratory acknowledges the critical requirement for sufficient biomaterial sampling and rapid handling. Portions of samples should be immediately snap frozen and stored appropriately at –80°C, to ensure their preservation for contemporary investigations, and to support research programmes. Furthermore, routine molecular diagnostics should be based on methods that are not sensitive to nucleic acid degradation, where possible. Finally, factors such as the nature of essential biomarkers, availability of cross‐disease methodologies (e.g. WGS) and cost/benefit considerations will also strongly influence the requirements for sample collection procedures, referral pathways and required biomaterials, for specific diseases.

Prompt liaison between local centres and NRCs is essential to ensure the timely and secure transit of biomaterials, with an infrastructure governance provided by the use of appropriate material transfer agreements. Once received by the NRC, robust tests performed in accredited laboratories, which first assess material quality and suitability for assessment, must be applied.^22^ Although not collected in our study, workflows for the adequate collection and storage of other biomaterials such as blood (for the assessment of tumour predisposition syndromes and germline genetic variation), CSF (for liquid biopsy approaches) and additional frozen tumour material for proteomics should be built into practice.

The introduction of MB molecular biomarkers into the CPR process both (i) enabled determination of associated risk‐status and (ii) highlighted requirements for the assessment and validation of any specific biomarker prior to its diagnostic use. Although specific biomarkers for any given disease will change over time – for instance, GAB1 and YAP1 IHC are now commonly used alongside β‐catenin to refine IHC‐based subgrouping of medulloblastoma^16^, ^19^—common underpinning principles may be established from our data.

For instance, in our study, assignment to the WNT biological subgroup was most reliable using multiple independent measures. *CTNNB1* mutation was both sensitive to most tumours and specific; however, a small proportion of *CTNNB1* wild‐type WNT cases alternatively harbour *APC* mutations.^23^ Molecular subgrouping by DNA methylation‐array was robust, sensitive and specific; methylation‐based methods are now entering the diagnostic repertoire but are not available in all institutions and require assessment at specialist centres.^24^ Therefore, independent assays and/or supporting measures are needed for the diagnostic assessment of molecular subgroup and, as such, this forms the current recommendation of the International Society for Paediatric Oncology (SIOP).^22^ Interpretation of β‐catenin IHC was highly variable in our blinded assessment of concordance between pathologists, supporting the requirement for objective molecular methods in this instance. The gold standard molecular analysis of *MYC* and *MYCN* status is iFISH, but failures were observed in our study. Improved success rates may have been observed if FFPE material had been used for iFISH, but nevertheless our experience suggests a requirement for collection of high‐quality frozen tissues and a role for additional methodologies. DNA methylation‐array approaches were highly specific but were prone to false negatives,^25^ possibly reflecting tumour heterogeneity, whereas MLPA methods commonly yielded false positives and were unsuitable for primary detection of gene amplifications.

Benchmarking and validation vs the current gold standard is, thus, required in the development/adoption of any new biomarker method. For example, array comparative genomic hybridization (aCGH) has been shown to be perform equivalently to iFISH for the detection of MYC/MYCN gene amplification.^26^ As novel (including NGS) technologies become available clinically for mutation, copy number and methylation assessment, robust validation against the current ‘gold standard’ becomes a key requirement.

Finally, reporting of molecular diagnostics to the local and/or treating centre must be in accordance with disease guidelines or the clinical study protocol. We (i) assessed diagnosis of our cohort to WHO (2016) standards and (ii) modelled enrolment from our cohort to the biomarker‐driven SIOP‐PNET5‐MB clinical trial, by reviewing our cohort against the tissue, NRC performance, clinical and molecular criteria required for trial recruitment. In our study, 23% (27/115) samples would have been ineligible due to delayed registration and turnaround times or low biomaterial quantity and quality; these factors can be managed and must be optimised to maximise populations eligible for recruitment to specific clinical studies. Of the 115 patients with complete data, 33 (29%) had altered risk status as a consequence of CPR assessments (i.e. assignment of *MYC*/*MYCN*, LCA, WNT and SHH‐*TP53* status).

## CONFLICT OF INTEREST

The authors declare no conflict of interests.

## AUTHOR CONTRIBUTIONS

Conception, design and study coordination: SCC, AM, BP, SB, SLN and SC. Experimentation: SC, SLN, DH, JCL and AS. Data analyses: DH, ECS and DW. Central pathology review: SBW, TSJ and AJ. Genetic assessment: Collection of patient data and clinical specimens: NB and GC. SC, SLN and SB. Manuscript writing: SCC, DH and SC. All authors contributed to and approved the final manuscript.

## ETHICAL APPROVAL

Human tumour samples were provided by the UK CCLG as part of CCLG‐approved biological study BS‐2008–12; informed consent was obtained from all subjects and investigations conducted with approval from Newcastle/North Tyneside Research Ethics Committee (study reference 07/Q0905/71).

## Supporting information

Supplementary Material

## Data Availability

The data that support the findings of this study are available from the corresponding author on reasonable request.
