# Supplementary material for: Advanced molecular pathology for rare tumours: A national feasibility study and model for centralised medulloblastoma diagnostics
Source: Neuropathol Appl Neurobiol. 2021 May 2;47(6):736–47. doi: 10.1111/nan.12716 (PMC8600954; doi:10.1111/nan.12716)

**Advanced molecular pathology for rare tumours: A national feasibility study and model for centralised medulloblastoma diagnostics**

## Crosier et al.

**Supplementary materials and methods**

*Logistical data collection*

Times taken for shipment, tissue processing and diagnostic assessment reporting (central pathology review and molecular testing) were recorded to inform logistical best practices to underpin upcoming trials and are listed in Table S1.

| **Parameter** | **Time from (days)** | **Time to (days)** |
| --- | --- | --- |
| Tissue processing and preparation | Receipt in NRC | Dispatch for cytogenetics |
| Analysis (iFISH) | Dispatch for iFISH | Receipt of iFISH results |
| Tissue preparation histology/IHC | Receipt in NRC | Dispatch for CPR |
| Central pathology review | Dispatch for CPR | CPR report |
| NRC performance | Receipt in NRC | Reporting to local centre |
| Local diagnosis | Date of surgery (DOS) | Date of local diagnosis |

**Table S1:** Logistical data recorded.

## Personnel availability and the requirement for technical IHC repeats or additional CPR were also recorded.

*FFPE histology*

A medulloblastoma diagnostic IHC panel was applied to pre-mounted slides with their appropriate control sections (Table S2) using the fully automated Ventana BenchMark XT IHC system and standard detection reagents ultraVIEW™ Universal DAB Detection kit (Ventana Medical Systems; Cat No: 760-500). This methodology incorporated antigen retrieval with Ventana ultra cell conditioning 1 and a haematoxylin counterstain (Ventana Medical Systems: Cat No: 950-224).

| **Antigen Source** | **Catalogue Dilution Clone** | | | **Control tissue** |
| --- | --- | --- | --- | --- |
|  | **Number** |  |  |  |
| INI-1 (BAF47) BD  Transduction Laboratories | 612111 | 1/100 | 25/BAF47 | Tonsil |
| β-catenin BD  Transduction Laboratories | 610154 | 1/100 | Clone14 | Colonic cancer |
| GFAP DAKO | M 0761 | 1/250 | 6F2 | Brain |
| Synaptophysin DAKO | M7315 | 1/100 | DAK-  SYNAP | Carcinoid |
| NFP DAKO | M0762 | 1/100 | 2F11 | Colon |
| Vimentin DAKO | M7020 | 1/250 | Vim 3B4 | Colon |
| Ki 67 DAKO | M7240 | 1/100 | MIB-1 | Tonsil |
| P53 DAKO | M7001 | 1/100 | DO-7 | Lymphoma |

**Table S2:** Medulloblastoma immunocytochemistry panel. GFAP; Glial Fibrillary Acidic Protein, NFP; Neurofilament Protein.

*Nucleic acid extraction*

DNA was extracted using the DNeasy Blood and Tissue Kit (Qiagen, Cat No. 69504; frozen tissue) and QIAamp DNA FFPE Tissue Kit (Qiagen, Cat No. 56404; FFPE tissue). DNA concentration was determined using the Qubit 2.0 Fluorometer (dsDNA BR assay; ThermoFisher Scientific). RNA was isolated from fresh frozen primary tumour samples using TRIzol, with subsequent clean-up using the RNeasy MinElute Cleanup Kit (Qiagen, Cat No. 74204). RNA Integrity Number (RIN) and concentration were determined using Bioanalyser analysis (RNA 6000 Nano assay; Agilent).

*Interphase fluorescence in-situ hybridisation (iFISH)*

Tissue was assessed for tumour content by H&E staining of 8μm frozen sections on

Superfrost plus slides and fixed in methanol/acetic acid and then air-dried.

iFISH was carried out following standard protocols using MYCN/LAF (Cytocell, LPS 009-SA) and MYC/IGH (Cytocell, LPS 035-SA) two-colour probe sets. Amplification was defined as more than 4-fold increase in the gene signal number compared with the reference probe in >5% of 100 non-overlapping nuclei.

*Molecular subgroup status*

DNA >11ng/µl in a volume <45 µl was suitable for 450K methylation array (Illumina). RNA quantity and quality was assessed by Agilent 2100 Bioanalyser and those RNAs with a RNA integrity number (RIN) exceeding 5 and >800ng were suitable for RNAseq based molecular subgrouping. Subgrouping according to methylation and expression profiles was achieved using established methods [1,2]. Consensus nonnegative matrix factorization (NMF) clustering of a primary medulloblastoma training cohort (n=132 methylation profiles, n=81 expression profiles) were used to define four subgroup-specific metagenes corresponding to the four consensus subgroups. A support vector machine (SVM) classifier to assign subgroup based on their projected metagene profiles, was developed using previously published methods [3]. Confidence of the classifier call made for these samples was assessed by repeated sampling of 80% of the training cohort to re-derive the classifier.

*Copy number estimation*

Copy-number assessment by MLPA (using SALSA reagents; MRC-Holland) for *MYC(N)* were as described [4] and measured relative to four independent reference loci (B2M, TBP, 7q31, and 14q22). Normal diploid control samples were used to define cutoffs for the detection of elevated copy numbers (>95% confidence interval of the normal distribution). Tumour samples showing reproducibly elevated copy numbers (in multiple replicates and versus three or more reference loci) were deemed to have copy-number elevation.

# Standard Operating Procedures (SOP) Sample receipt and handling by NRC

1. Local centre contacts will inform you of newly diagnosed medulloblastoma patient this is usually the local delegated individual coordinating tissue banking and consent:
   - Ensure case is fully reported by local pathologist.
   - Confirm CCLG Biobank consent.
   - Ensure both frozen and FFPE are available to send.
   - Ensure local centre has allocated a CCLG tissue bank number. Liaise with both the delegated local consent/tissue banking coordinator and laboratory sender contact. For samples from Newcastle – Contact research Nurse- to confirm consent has been given for trial inclusion. Research Nurse will confirm patient details. For tissue release contact Courier Booking Promptly arrange CRYOPDP courier at convenient time avoiding sample collections on weekends.
   - Approved courier contacted via customer service, quote appropriate account number
   - Request CRYOPDP collection of non-hazardous CAT B Frozen tissue (1 aliquot) and 1 x FFPE block in same package small dry ice package is sufficient, FFPE and paperwork usually sent in separate envelope on top of inner box to avoid direct contact with dry ice.
   - Request next day delivery, possible to request pre-10 am if particularly urgent.
   - Airway bills/tracking number will be provided by CRYOPDP PDP to the sender.
   - Sender adds frozen sample to the dry ice, with the paperwork and FFPE block added to the packaging.
2. Sample Transfer
   - E-mail trial registration form to sender (Appendix I).
   - Remind sender, courier provides all dry ice/packaging and only requires addition of sample.
   - Ensure they are sending frozen, FFPE and copy of pathology report.
3. Tissue receipt

- When tissue is received confirm receipt by e-mail to sender, ensure all tissue is from the correct patient by cross-checking lab number and tissue bank number present on the samples with the Appendix I form provided.
- Allocate trial number.
- Ensure all required information is present on Appendix I form, especially tissue bank number to commence preparing tissue.

1. Frozen Tissue Preparation
   - Cut one 7µm frozen section to send to reviewing pathologist to assess tumour cell content.
   - From the exposed face prepare eight touch-preps on superfrost plus adhesive slides.
   - Fix touch-preps in 3:1 99% IMS to acetic acid (45ml 99% IMS and 15ml acetic acid).
   - Send Touch preparations to cytogenetics with appropriate trial form (Appendix II)

*Note: Any queries regarding medulloblastoma and malignancy cytogenetics should be directed to the study mailbox which is regularly checked.*

- - Transfer remaining frozen tissue to a cryovial labelled with trial number, tissue bank number and local centre. Store at -80oC.

1. FFPE Tissue preparation

Note: H/E and beta-catenin required as an absolute minimum however review outcome may be limited with small sample types.

- - Cut 20 x 5 µm tissue sections on super frost plus adhesive slides as per the section list:

HE-1#

Reticulin GFAP#

SYNAPTOPHYSIN NFP

Ki-67

Vimentin INI-1#

β−catenin# P53#

9x USS (if sufficient tissue) HE -2 for TMA mark-up

- - If FFPE is tiny prioritize essential slides labelled with (#).
  - If sufficient tissue is available, take tissue curls (5 x 20µm curls to a 1.5ml cryovial) prior to HE-2 for frozen storage and transfer to -80oC archive
  - Transfer curls to frozen archive and store at -80oC
  - Label all slides/vials with trial number, unstained sections are stored in CCLG file in archive room.

1. IHC Staining

- The full IHC panel was applied with their appropriate control sections (listed above).
- Panel stained on automated Ventana BenchMark XT IHC system using standard detection reagents ultraVIEW™ Universal DAB Detection kit (Ventana Medical Systems; Cat No: 760-500).

1. Haematoxylin and Eosin – paraffin sections [5].
   - De-wax sections in xylene **10 minutes.**
   - Sections pass down IMS alcohol gradient to water (absolute IMS,95% IMS, 70% IMS, water).
   - Haematoxylin **4 minutes.**
   - Rinse in tap water **30 seconds.**
   - Differentiate in acid alcohol **2 seconds.**
   - Blue in Scott’s tap water **30 seconds.**
   - Rinse in water **30 seconds.**
   - Eosin **3 minutes.**
   - Dehydrate up IMS gradient clear in xylene and coverslip. Results: Nuclei deep blue

Muscle deep pink Collagen pink

Red blood cells orange/red

Other substances shades of pink and red.

1. Gordon and Sweets reticulin Stain [5]– paraffin Sections

Reticulin fibres are very thin delicately woven strands of type III collagen which are highly ordered and provide a support network within tissue. Many of these types of collagen are combined with carbohydrate and react with silver type stains as in the method described. Reticulin is useful for medulloblastoma diagnotics to assess tumour architecture and is especially diagnostic for the demonstration of nodules in nodular desmoplastic tumour sub-types.

- - The method is controlled by a known positive tissue control such as liver.
  - De-wax sections in xylene **10 minutes.**
  - Sections pass down IMS alcohol gradient to water (absolute IMS,95% IMS, 70% IMS, water).
  - Oxidise with 1% potassium permanganate (KMnO4) in 0.25% sulphuric acid (H2SO4) **5 minutes.**
  - Wash in tap water **30 seconds.**
  - Apply 5% oxalic acid (CH2O4.2H20) **2 minutes.**
  - Wash in tap water **30 seconds.**
  - Apply 4% iron alum (FeNH4(SO4)2 .12H2O) **2 minutes.**
  - Wash in distilled water **30 seconds.**
  - Filter on ammoniacal silver solution **1 minute.**

Ammoniacal silver solution:

To 5ml of 10% silver nitrate (AgNO3) add drops of ammonia (NH3) until a precipitate forms and just dissolves, avoid any excess of ammonia. Add 5ml of 3% sodium hydroxide (NaOH) and re-dissolve the resultant precipitate with more ammonia until the solution retains faint trace of opalescence. Make up to 50ml with distilled water, filter and stain slides. Store for up to 1 week at 40C in fridge.

- - Rinse rapidly in distilled water and reduce in 10% formalin agitating. **3 minutes.**
  - Wash well in tap water **1 minute.**
  - Tone in 0.2% gold chloride **10 seconds.**
  - Wash in tap water **30 seconds.**
  - Fix in “hypo” 2%sodium thiosulphate solution (Na2O3S2.5H20).
  - Wash in tap water **30 seconds.**
  - Counterstain in 1% neutral red.
  - Wash in water **30 seconds.**
    - Dehydrate up IMS alcohol gradient as above, clear in xylene and coverslip. Results:

Reticulin fibres –black Nuclei – red

1. Central Pathology Review

- Allocate a reviewing neuropathologist – e-mail/call them to ensure availability to review the case. Send prepared material via registered post, include CPR form (Appendix III) and a copy of the local centre pathology report.
- Central review pathologist will return prepared material.
- Prepared material filed in MB research archive RVI

1. Result Collation
   - Advanced Research Neuropathology Biomedical scientist to collate results.
   - Follow up with CPR Neuropathologist within 5 days.
   - Reviewer will fax/email CPR form. Cytogenetics will post and email full report to Advanced Research Neuropathology Biomedical Scientist.
   - Ensure all information collated and logged in trial database.
2. Local Centre Confirmation.

- Confirm review outcome to local centre.
- E-mail confirmation of results form to local centre reporting pathologist by completing review confirmation form (Appendix IV).

1. Update CCLG

- Inform CCLG that tissue has been submitted for the trial.
- Email tissue bank number, local centre, date of dispatch and receipt.

**References**

1. Hovestadt V, Remke M, Kool M, Pietsch T, Northcott PA, Fischer R, et al. Robust molecular subgrouping and copy-number profiling of medulloblastoma from small amounts of archival tumour material using high-density DNA methylation arrays. Acta Neuropathol. 2013; 125: 913-916
2. Brunet JP, Tamayo P, Golub TR, Mesirov JP. Metagenes and molecular pattern

discovery using matrix factorization. Proc NatL Acad Sci. 2004;101(12): 4164-9

1. Schwalbe EC, Williamson D, Lindsey JC, Hamilton D, Ryan SL, Megahed H, et al. DNA methylation profiling of medulloblastoma allows robust subclassification and improved outcome prediction using formalin-fixed biopsies. Acta Neuropathol. 2013; 125(3):359-71.
2. Hill RM, Kuijper S, Lindsey JC, Petrie K, Schwalbe EC, Barker K, et al. Combined MYC and P53 Defects Emerge at Medulloblastoma Relapse and Define Rapidly Progressive, Therapeutically Targetable Disease. Cancer Cell. 2015; 27(1):72-84.
3. Suvarna KS, Layton C, Bancroft JD. Bancroft's Theory and Practice of Histological Techniques: 8^th^ Edition Elsevier; 2019: https://doi.org/10.1016/C2015-0-00143-5.

# Appendix I: Patient request and registration form.


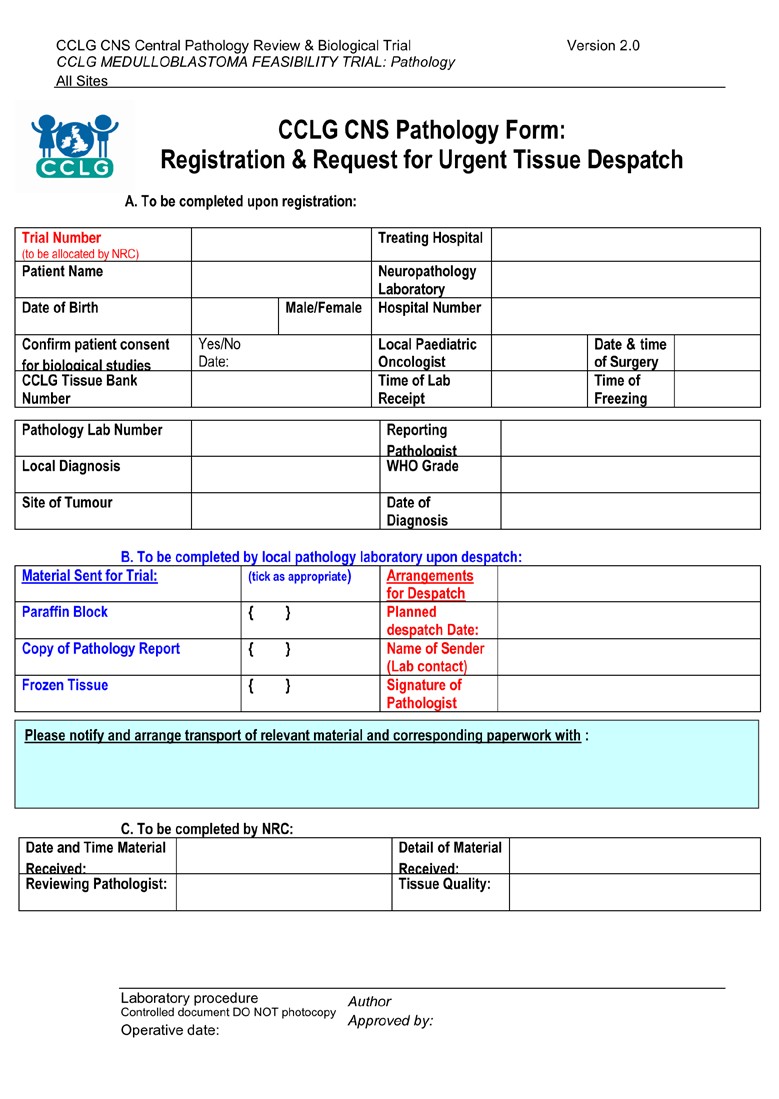


**Appendix II: iFISH and cytogenetics request form**


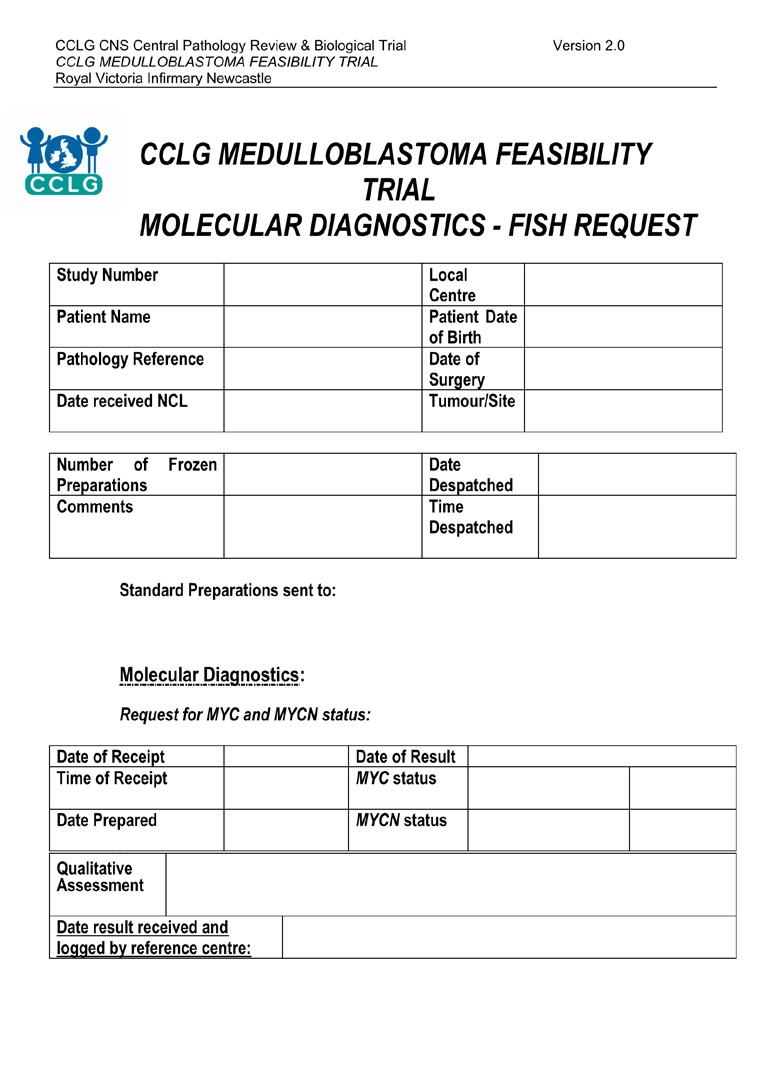


**Appendix III: Central pathology review form**


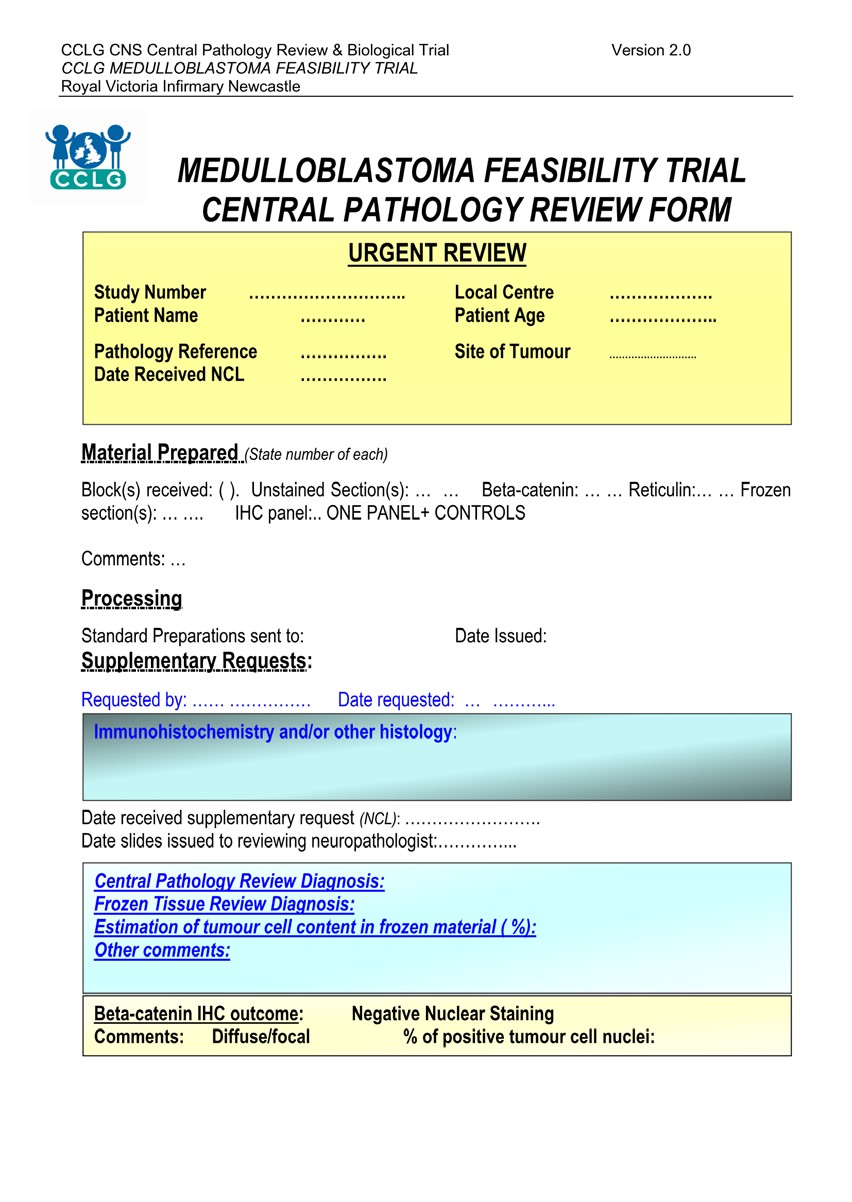


**Appendix IV: Review and molecular analysis results confirmation form**


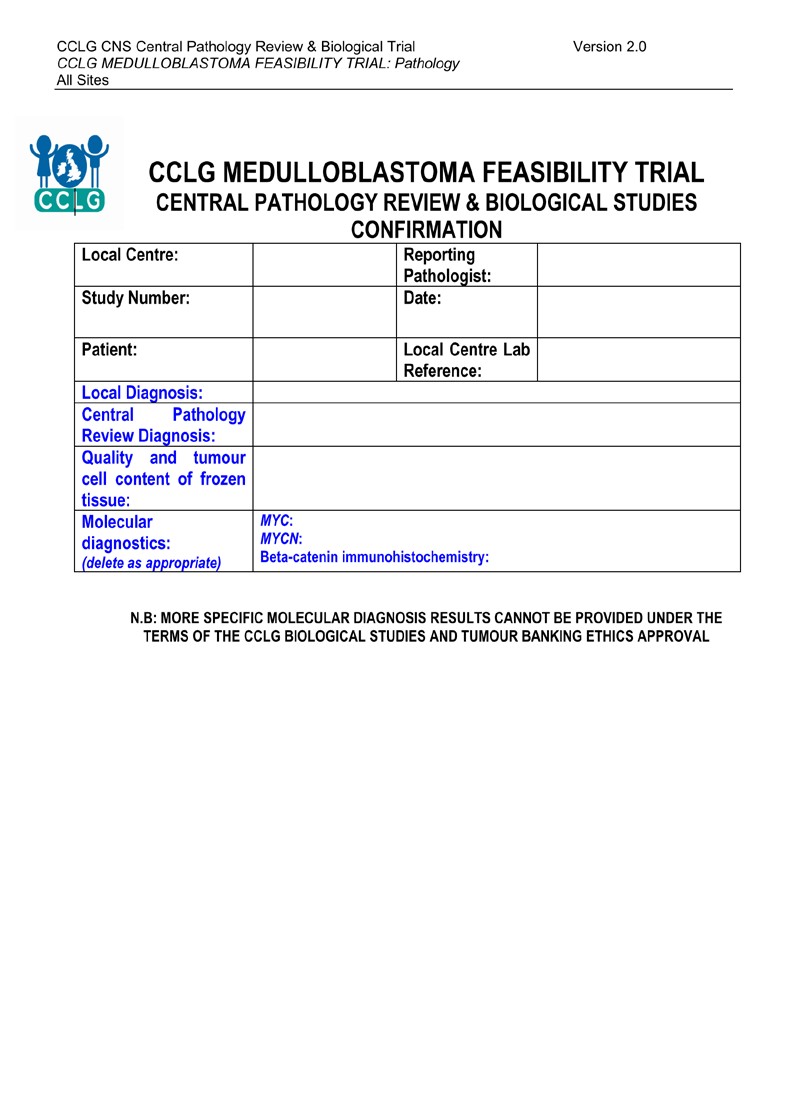

Supplement: Supplementary file 1 — Supplementary Material [file NAN-47-736-s001.docx]
